# *Escherichia coli* outer membrane vesicles: Bacterial virulence, antibiotic resistance, host interactions, and applications in biomedical platforms

**DOI:** 10.1080/21505594.2026.2707805

**Published:** 2026-07-21

**Authors:** Bo Yan, Siyi Cai, Yujin Wang, Qingwen Li, Wenqiang Guo, Xiaoliu Huang, Elvis Agbo, Xianyun Xu, Kunhao Qin, Qiang Fu, Yafang Ding

**Affiliations:** a Health Science Center, Jinggangshan University, Ji’an, China; b Jiangxi Province Key Laboratory of Organ Development and Epigenetics, Clinical Medical Research Center, Affiliated Hospital of Jinggangshan University, Ji’an, China; c Department of Clinical Laboratory, Ji’an Central People’s Hospital, Ji’an, China

**Keywords:** Outer membrane vesicles, Escherichia coli, virulence, antibiotic resistance, immune modulation, biotechnological applications

## Abstract

*Escherichia coli* is a clinically significant Gram-negative pathogen, and the emergence of multidrug-resistant and highly virulent strains poses a grave threat to public health. Outer membrane vesicles (OMVs) are spherical nanostructures secreted by Gram-negative bacteria and composed of the outer membrane and a suite of encapsulated bioactive components, including proteins, lipopolysaccharides, and nucleic acids. Research indicates that OMVs play pivotal roles in pathogenic mechanisms, antimicrobial resistance transmission, and host-pathogen interactions. This review systematically elucidates the biogenesis mechanisms and molecular composition of *E. coli* OMVs while thoroughly examining their core biological functions in virulence, resistance, and immune modulation. Additionally, this review evaluates the translational potential of OMVs in vaccine development, biomolecular delivery, and tumor therapy. In summary, a comprehensive understanding of the biological properties and functions of *E. coli* OMVs is crucial for developing innovative OMV-based strategies for infection control, diagnosis, and therapy, thereby providing valuable directions for future research.

## Introduction

*Escherichia coli* is a Gram-negative bacterium commonly found as a commensal in the intestines of humans and other warm-blooded animals. However, certain strains are pathogenic and can cause severe diseases such as diarrhea, urinary tract infections, intestinal infections, and sepsis, posing a significant risk to women, the elderly, and immunocompromised individuals [1, 2]. The emergence of highly virulent and multidrug-resistant *E. coli* strains in recent years has intensified the public health threat posed by this pathogen. Of particular concern are extended-spectrum β-lactamase (ESBL)-producing and fluoroquinolone-resistant *E. coli* strains, as severe infections caused by these strains are associated with high mortality rates attributable to antimicrobial resistance [3–5]. The World Health Organization’s 2023 Bacterial Priority Pathogens List explicitly identifies ESBL-producing *E. coli* as a critical priority pathogen, highlighting the urgent need for new antibacterial agents. These strains exhibit widespread resistance to first-line antibiotics like ceftriaxone and are frequently transmitted in both community and hospital settings [6]. According to 2024 WHO antimicrobial resistance surveillance data, drug-resistant *E. coli* was the leading cause of deaths from resistant Gram-negative bacterial infections in low- and middle-income countries [7]. Among these, high-risk multidrug-resistant strains, epitomized by the ST131 clone (particularly the H30R and H30Rx subclones), are especially concerning. The prevalence of these ST131 lineages among drug-resistant *E. coli* isolates often reaches 50%–70%, and infections caused by them are associated with significantly higher mortality rates than those caused by susceptible strains [4, 8]. These grave realities underscore the significant threat that drug-resistant *E. coli* infections pose to global health.

The secretion of extracellular vesicles (EVs) is a critical mechanism through which organisms interact with their environment. Studies have shown that bacteria achieve this by releasing nanoscale spherical vesicles, a capacity shared by both Gram-positive and Gram-negative bacteria [9]. Due to the absence of an outer membrane, EVs secreted by Gram-positive bacteria are generally designated as membrane vesicles (MVs) [10], whereas those secreted by Gram-negative bacteria are classified as outer membrane vesicles (OMVs) [11]. These vesicles typically range from 20 to 400 nm in diameter and are enriched with bioactive components, including lipopolysaccharides (LPS), phospholipids, proteins, toxins, and genetic material. OMVs play crucial roles in the delivery of virulence factors, biofilm formation, the dissemination of antibiotic resistance, nutrient transport, interspecies competition, horizontal gene transfer, and host-pathogen immune interactions [9, 12–14]. Owing to their cargo of bacterial antigens and diverse pathogen-associated molecular patterns (PAMPs), coupled with their ability to modulate immune responses, OMVs have emerged as a pivotal platform for biotechnological applications, with OMV-based vaccines representing the most advanced area [15–18]. Furthermore, OMVs are being explored as potent drug delivery vehicles for integrating immunotherapy with chemotherapy or photodynamic therapy to enhance anti-tumor efficacy [19, 20]. Of note, drug resistance constitutes a major challenge in treating both cancer and bacterial infections. Research indicates that engineered EVs (such as exosomes) can effectively overcome this barrier; for instance, by delivering resistance reversal agents or siRNA to silence resistance-associated genes, thereby significantly sensitizing tumor cells to chemotherapeutic drugs [21]. Translating this strategy to bacterial infections, OMVs derived from Gram-negative bacteria, owing to their inherent membrane fusion properties, high loading capacity, and immunomodulatory functions, hold promise for delivering antibiotic adjuvants, targeting resistant bacterial biofilms, or modulating the infection microenvironment, thereby offering innovative strategies to combat the growing threat of bacterial resistance.

**Figure 1.**
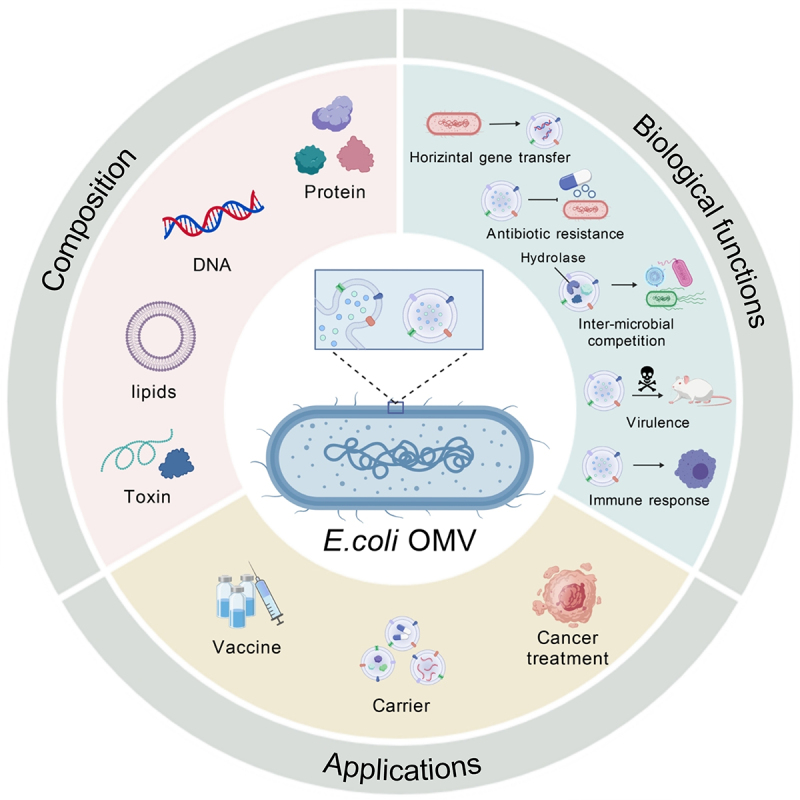
Composition, biological functions, and potential applications of *E. coli* OMVs. The composition of *E. coil* OMV includes proteins, nucleic acids, lipopolysaccharides, and lipids. *E. coil* OMV plays a significant role in horizontal gene transfer, antibiotic resistance, inter-microbial competition, virulence, and immune response. *E. coli* OMVs exhibit versatile applications in the biomedical field, encompassing vaccines, cancer therapy, and biomolecule delivery. Created with BioGDP.com [32].

While OMV biology is conserved across Gram-negative bacteria, prioritizing *E. coli* offers three distinct and strategic advantages. First, its unrivaled genetic toolbox (including CRISPR-Cas systems and diverse plasmid vectors) allows for precise dissection of biogenesis pathways and cargo-sorting mechanisms, establishing a molecular blueprint that can be extrapolated to less tractable pathogens [22–24]. Second, the coexistence of highly pathogenic (e.g. ST131, EHEC O157) and probiotic (e.g. Nissle 1917) strains within the same species provides a unique isogenic background to compare virulence vs immune-modulation functions of OMVs – a natural experiment impossible with other bacteria [25, 26]. Third, and most critically, the emergence of colistin resistance and ESBLs in *E. coli* has created a public health emergency [27–31], understanding how OMVs disseminate these genes is not merely academic but a prerequisite for designing interventions. Therefore, this review systematically summarizes recent advances in *E. coli* OMV research, covering their biogenesis, molecular composition, biological functions, and their roles in mediating antibiotic resistance and modulating host immune responses during infection (Figure 1). Additionally, it discusses the potential applications of *E. coli* OMVs in novel vaccine design, drug delivery systems, and tumor therapy.

## Biogenesis and molecular composition of OMVs

**Figure 2.**
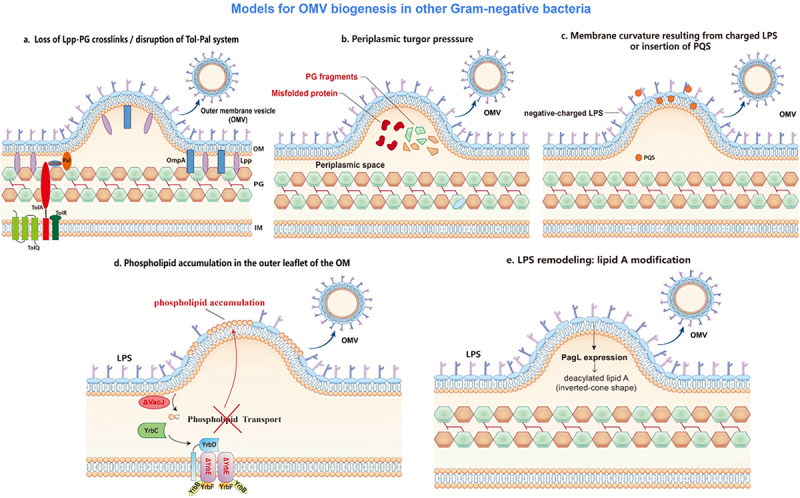
*E. coli* OMV biogenesis models: a. Reduced cross-linking between the outer membrane and peptidoglycan layer promotes outer membrane vesiculation by inducing local membrane instability. b. Accumulation of periplasmic contents, such as peptidoglycan fragments, exerts swelling pressure on the outer membrane, triggering vesicle formation. c. Asymmetric expansion of the outer leaflet of the outer membrane, driven by biomolecule insertion, induces membrane curvature and vesicle budding. d. Disruption of the VacJ/YRB ABC transport system leads to phospholipid accumulation in the outer membrane, resulting in hypervesiculation. e. Lipopolysaccharide remodeling alters membrane charge and fluidity, enhancing outer membrane curvature and vesicle production. Reprinted from ref [35]. The figure originates from an article published under the Creative Commons Attribution 4.0 International License.

In 1965, researchers first observed that a lysine auxotroph of *E. coli* released membrane-derived vesicles, primarily composed of LPS, under lysine-restricted culture conditions [33]. Subsequent decades of research revealed these vesicles’ high structural and compositional similarity to the outer membrane, providing crucial insights into their biogenesis [11, 34]. OMVs primarily form via budding or outward blebbing of the OM, a process analogous to the biogenesis of exosomes in eukaryotes. This mechanism enables OMVs to deliver virulence factors, periplasmic and cytoplasmic proteins, and genetic material into host cells. Currently, OMVs biogenesis is attributed to five major theoretical models: (a) Dissociation of OM – peptidoglycan linkage: In *E. coli*, the lipoprotein Lpp, which anchors the OM to the peptidoglycan layer, is incorporated into vesicles during OMVs formation. This incorporation locally weakens the OM-peptidoglycan linkage, thereby driving OMVs release by facilitating outer membrane expansion and curvature [9]. Additionally, disruption of the Tol-Pal system, which comprises the inner membrane proteins TolQ, TolR, and TolA, the periplasmic protein TolB, and the outer membrane lipoprotein Pal, compromises OM-peptidoglycan anchoring and induces a hypervesiculation phenotype (Figure 2a). (b) Accumulation of periplasmic components: The aggregation of peptidoglycan fragments or misfolded proteins in the periplasm generates turgor pressure against the OM, triggering vesicle formation (Figure 2b) [35]. (c) Bilayer Coupling Model: The insertion of certain molecules into the outer leaflet alters local membrane curvature. An expansion rate of the outer leaflet that surpasses that of the inner leaflet creates asymmetry, inducing outward budding. However, this mechanism has primarily been reported in pathogens producing the pseudomonas quinolone signal and has not yet been documented in *E. coli* (Figure 2c) [36]. (d) Dysfunction of the VacJ/YRB ABC transporter system: This system maintains lipid asymmetry by retrograde transporting phospholipids from the OM to the inner membrane. Its impairment causes phospholipid accumulation in the outer leaflet, leading to excessive OMVs secretion (Figure 2d) [37]. (e) LPS lipid A deacylation-mediated OM remodeling: Deacylation of lipid A increases membrane curvature, thereby promoting OMVs formation (Figure 2e) [38].

Despite advances in elucidating these mechanistic models, their universality in *E. coli* OMVs biogenesis requires further validation. OMVs production occurs throughout all bacterial growth stages, though their yield is modulated by diverse environmental factors, including temperature, osmotic pressure, culture medium status, extracellular ion concentrations, and oxidative stress [13, 39]. Genetically, deletion of the *rfb* gene cluster (responsible for O-antigen synthesis) reduces outer membrane fluidity and causes cytoplasmic protein leakage, consequently increasing OMVs production and altering its composition [40]. Furthermore, exposure to specific antibiotics like ciprofloxacin and meropenem directly alters OMVs morphology and quantity [41]. Under nutritional stress such as iron limitation, uropathogenic *E. coli* specifically produces OMVs with larger diameters and higher concentrations [42, 43]. In summary, *in vitro* studies have established that OMVs biogenesis and cargo are co-regulated by a combination of physical, chemical, genetic, and nutritional factors. However, a comprehensive understanding of their pathogenic functions during infection requires systematic analysis of their dynamic secretion mechanisms within the host and the interplay among these factors.

*E. coli* OMVs contain a diverse array of functional proteins and exhibit substantial compositional heterogeneity. This heterogeneity is co-regulated by extraction methods, bacterial genetic background, and growth environment. Using liquid chromatography-mass spectrometry, González et al. identified 282 and 215 distinct proteins in OMVs from the U144 strain cultured in LB medium and artificial urine, respectively [44]. Cytoplasmic proteins constituted the largest proportion (LB: 36.9%; urine: 37.2%), followed by inner membrane proteins (LB: 17.4%; urine: 21.4%) and outer membrane proteins (LB: 14.9%; urine: 17.7%). In contrast, Zanella et al. identified only 81 OMV proteins using two-dimensional gel electrophoresis, highlighting the significant impact of methodological sensitivity on the reproducibility of proteomic profiles [45]. Genetic background also significantly shapes the OMV proteome. For instance, deletions in LPS synthesis-related genes (*rfaC*, *rfaG*, *rfaL*) specifically alter the expression of stress- and metabolism-associated proteins. In Δ*rfaC* and Δ*rfaG* mutants, metabolic proteins (GalF), protein-folding chaperones (GrpE, ClpX), and fatty acid metabolism-related proteins (AccA, FabB) are significantly upregulated, whereas membrane transporters (PspA, YdiY) and ribosomal proteins (RpsT, RpmB) are markedly downregulated in Δ*rfaL* mutants. These findings indicate that LPS synthesis defects promote bacterial adaptive evolution by rewiring metabolic and stress response pathways [46]. Similarly, environmental stress remodels the OMV proteome. Under iron-deficient conditions, extraintestinal pathogenic *E. coli* (ExPEC) exhibits increased OMV production, with significant enrichment of iron acquisition-related proteins, such as the siderophore receptors CirA and FepA, siderophore synthesis enzymes EntB and IucD, and the iron storage protein FtnA [47]. This phenomenon implies that OMVs serve as an adaptive strategy for bacteria to maintain iron homeostasis under nutritional stress.

Lipids are also essential components of *E. coli* OMVs, with phospholipids and LPS forming the core structural elements, and their composition is closely linked to bacterial environmental adaptability [48, 49]. The phospholipids in OMVs are predominantly phosphatidylethanolamine, phosphatidylglycerol, and cardiolipin, mirroring the composition of the bacterial outer membrane [50]. Moreover, OMVs are enriched in saturated fatty acyl chains, which enhances their stability in harsh environments. Nevertheless, the current lack of lipidomics-based quantitative studies on OMV phospholipids impedes a deeper understanding of lipid asymmetry and vesicle biogenesis. LPS is another critical component of OMVs, and its structural heterogeneity directly influences bacterial pathogenicity. Pathogenic strains (e.g. EPEC, ETEC, EHEC) express LPS with long-chain O-antigens that carry epitopes mimicking human Lewis blood group antigens. These epitopes are based on two distinct Gal-GlcNAc backbones: Type I, Gal-β-(1,3)-GlcNAc, which is modified to form Lewis a (Le^a^) and Lewis b (Le^b^) antigens; and Type II, Gal-β-(1,4)-GlcNAc, which forms Lewis ×(Le^x^) and Lewis y (Le^y^) antigens upon fucosylation [51–53]. In contrast, laboratory strains typically possess truncated or absent O-antigens. These structural differences not only determine serotype classification but also facilitate immune evasion through molecular mimicry of host antigens, thereby providing a molecular basis for pathogenic colonization. Consequently, the dynamic shifts in OMV lipid composition, in conjunction with pathogen-specific modifications of LPS, collectively regulate their environmental adaptability and pathogenic mechanisms. This provides novel opportunities for vaccine design and anti-infection strategies that target lipids or LPS.

Genetic materials, including extracellular DNA and small non-coding RNAs, have been identified in OMVs derived from diverse Gram-negative bacteria [54–56]. Pathogenic *E. coli* OMVs serve as versatile nanocarriers that package virulence factors, antibiotic resistance elements, and nucleic acids, thereby participating in interspecies communication and host invasion. Virulence factor composition exhibits remarkable strain specificity. For example, ETEC OMVs are enriched with heat-labile enterotoxin (LT), the EtpA adhesin, and colonization factors [57], whereas EHEC OMVs predominantly carry Shiga toxins (Stx1, Stx2a), the cytolethal distending toxin variant CdtV, and hemolysin [58]. Furthermore, Berzosa et al. simultaneously detected multiple virulence genes (e.g. *eltAB*, *cfaI*, *cs3*) alongside non-classical virulence proteins within ETEC OMVs [59], suggesting that OMVs can mediate pathogenicity through synergistic protein and nucleic acid mechanisms.

Regarding antibiotic resistance, subinhibitory concentrations of antibiotics induce the targeted enrichment of resistance elements within OMVs. For example, ampicillin treatment tripled the β-lactamase content in OMVs from extraintestinal pathogenic *E. coli* (ExPEC) ST131, significantly enhancing their protective effect on susceptible bacteria [60]. Proteomic analysis further revealed that the differentially expressed proteins in these OMVs were predominantly involved in resistance, stress response, and metabolic processes. Similarly, in a *Galleria mellonella* infection model, OMVs carrying the NDM-1 resistance gene protected carbapenem-susceptible *E. coli* and *Pseudomonas aeruginosa*, functioning as “mobile resistance reservoirs” that sustain the transmission of resistant phenotypes [61]. This protective effect involves a dual mechanism: the immediate degradation of antibiotics by enzymes like β-lactamases, and the sustained dissemination of resistance via plasmid-borne genes such as *tet*(X4) and *mcr* [62]. By coupling the delivery of virulence factors with the spread of resistance through spatiotemporally dynamic molecular sorting mechanisms, OMVs provide a mechanistic rationale for developing novel anti-infective strategies that target their core components or block membrane fusion.

In summary, *E. coli* OMVs are nanoscale secreted vesicles loaded with a selective cargo of proteins, virulence factors, resistance-associated molecules, lipids, LPS, and genetic material. The composition and abundance of these vesicular components are regulated by the strain’s genetic background, growth environment, and antibiotic exposure. Through the targeted delivery of effector molecules, OMVs directly participate in key pathogenic processes, including host invasion, horizontal resistance transmission, and environmental adaptation. Although their fundamental composition is relatively well-characterized, the condition-dependent mechanisms governing molecular sorting and the causal relationships with pathogenic phenotypes remain central challenges in current research. Therefore, systematically elucidating the biological functions of OMVs – particularly the specific action mechanisms of their molecular cargo in host-microbe interactions – is essential for a comprehensive understanding of OMV-mediated pathogenicity and for developing effective countermeasure strategies.

## Biological functions of E. coli OMVs

The diverse biomolecules and unique structure of OMVs underpin their broad spectrum of physiological and pathological functions. Specifically, studies have confirmed their significant roles in pathogenic mechanisms, modulating host inflammatory and immune responses, disseminating antibiotic resistance, facilitating interspecies interactions, and their implication in bacterial-induced male infertility [63–65].

### The role of E. coli OMVs in the pathogenesis of pathogen infection

Recent studies have indicated that OMVs derived from pathogenic *E. coli* are closely implicated in its pathogenic mechanisms [13, 66–68]. They function as a sophisticated, non-classical secretion system that achieves targeted delivery and functional synergy of virulence factors, thereby amplifying pathogenicity beyond the capabilities of freely secreted molecules. The pathogenic specificity of OMV delivery begins with precise host cell recognition. This is governed by a molecular instruction system on the OMV surface. Of particular importance among these are LPS O antigens and outer membrane adhesins. For instance, in ETEC, the EtpA adhesin enriched on OMVs directly mediates binding to host intestinal epithelial cells [57]. The absence of O antigens, as shown in isogenic mutants, significantly impairs OMV attachment and subsequent internalization by host cells, underscoring their role as critical recognition hubs [69, 70]. Following attachment, pathogenic *E. coli* OMVs are primarily internalized via clathrin-mediated endocytosis [9, 71]. Notably, the invasion efficiency is strikingly dependent on the vesicle cargo; OMVs from pathogenic strains, laden with toxins like LT or Cif, exhibit far greater internalization rates than those from nonpathogenic counterparts [72, 73]. This indicates that virulence factors themselves may actively participate in orchestrating the entry process.

Once internalized, OMVs provide a protected microenvironment that facilitates the functional synergy of co-packaged toxins, leading to exacerbated tissue damage [74, 75]. A paradigmatic example is the co-delivery of LT and the pore-forming toxin ClyA by ETEC OMVs [76]. These effectors operate in a concerted, multi-hit mechanism: (1) LT-driven secretion: LT catalyzes a sustained increase in intracellular cyclic AMP, leading to the dramatic efflux of chloride ions and water into the intestinal lumen, causing profuse watery diarrhea. (2) ClyA oligomerizes within the host cell membrane to form pores, disrupting ionic balance and critically compromising tight junction integrity. This breach in the epithelial barrier not only exacerbates fluid loss but also facilitates paracellular translocation of bacteria and other luminal contents, potentiating inflammation and prolonging disease. This synergy demonstrates how OMVs transform individual toxin actions into a coordinated pathogenic assault. While other pathogens (e.g. *V. cholerae*, *H. pylori*) also deliver toxins via OMVs, the well-characterized, multi-toxin synergy within single *E. coli* OMVs provides the clearest example of “pathogenic payload packaging,” offering a quantitative model for studying bacterial warfare strategies. Furthermore, the exogenous addition of purified wild-type OMVs can often restore or even enhance the virulence of these deficient cultures, confirming OMVs as active virulence vehicles. Similarly, Krsek et al. demonstrated that OMVs of EHEC O157 function as a co-delivery vehicle for multiple critical toxins, including Stx2a, CdtV, and EHEC-Hly [77]. Following internalization, these toxins dissociate from the vesicles and act synergistically on endothelial cells: Stx2a inhibits protein synthesis, CdtV induces cell cycle arrest, and EHEC-Hly impairs mitochondrial function. This coordinated multi-toxin attack ultimately triggers endothelial apoptosis and promotes microvascular thrombosis. Further investigations confirm that even when OMVs shed part of their toxin cargo during translocation, they maintain considerable cytotoxicity after crossing cellular barriers, underscoring their role as integrated virulence units. In contrast, bacterial strains or their OMVs lacking key virulence factors (e.g. Stx2a) preserve translocation ability but show substantially diminished pathogenic potential, indicating that the specific combination of virulence factors carried by OMVs constitutes the material basis for their enhanced pathogenicity. These functional loss-and-gain experiments unequivocally position OMVs as central executors of *E. coli* pathogenesis, rather than passive by-products.

### E. coli OMVs mediate host inflammatory and immune responses

**Figure 3.**
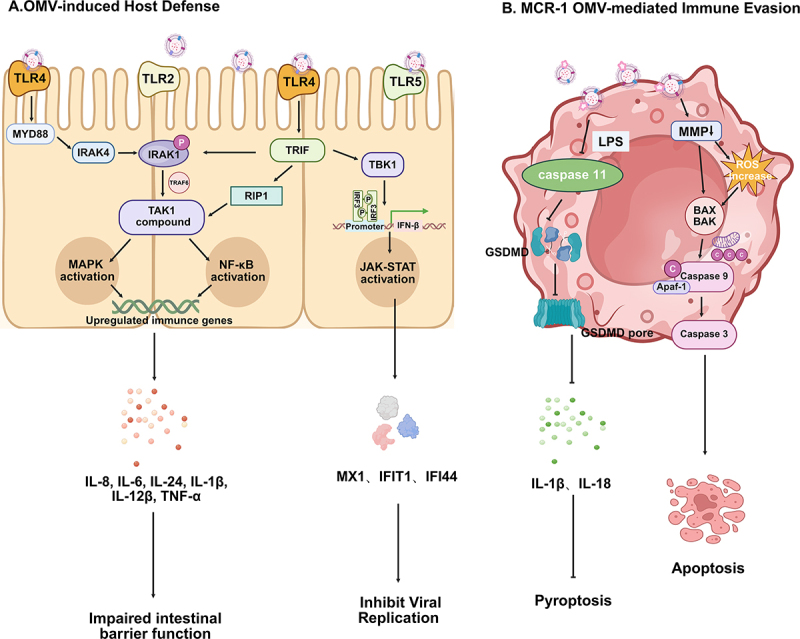
Activation of host inflammatory and immune responses by *E. coli* outer membrane vesicles. (A) *E. coli* OMV-induced host defense. OMVs carry pathogen-associated molecular patterns such as LPS, outer membrane proteins, which are recognized by a broad range of host Toll-like receptors including TLR2, TLR4, TLR5, and others, leading to the activation of distinct downstream signaling pathways, driving both a robust proinflammatory response and the induction of antiviral defenses. (B) MCR-1-containing *E. coli* OMVs mediate immune evasion. The presence of the MCR-1 gene modifies the lipid a structure of LPS on OMVs, thereby reducing their recognition by TLR4 and dampening the host inflammatory response. Concurrently, these modified OMVs induce mitochondrial dysfunction and reactive oxygen species accumulation in macrophages, ultimately leading to apoptotic cell death. Drawn by BioRender.

Pathogenic *E. coli*-derived OMVs carry multiple PAMPs that are recognized by host pattern-recognition receptors, thereby initiating both pro-inflammatory responses and immunomodulatory programs. As demonstrated in Figure 3a, upon OMVs recognition, epithelial and immune cells-the first line of mucosal defense-orchestrate downstream signaling pathways that regulate cytokine release, the balance between apoptosis and proliferation, and bridge the adaptive immune response [78, 79]. Behrouzi et al. delineated the divergent immunostimulatory effects of pathogenic vs nonpathogenic *E. coli* OMVs [80]. LPS in pathogenic OMVs, upon TLR4 recognition, potently activates the NF-κB pathway, inducing high expression of inflammatory cytokines (e.g. IL-6, TNF-α, IL-1β), type I interferons, and chemokines (e.g. IL-8, CXCL1). This robust inflammatory response disrupts epithelial tight junctions, thereby compromising intestinal barrier function. In contrast, nonpathogenic OMVs elicit only mild TLR activation and anti-inflammatory responses, conducive to homeostasis maintenance. IL-17 levels remained unaltered by stimulation with either OMV type, potentially due to negative feedback regulation by type I interferons. Furthermore, Bierwagen et al. recently discovered that pathogenic *E. coli* OMVs can activate an antiviral protective state via the TLR4–TRIF branch [81]. This mechanism relies on sustained phosphorylation of IRF-3, which promotes IFN-β synthesis. Subsequent JAK-STAT signaling upregulates interferon-stimulated genes (e.g. MX1, IFIT1, IFI44), thereby significantly suppressing influenza virus replication. Notably, this antiviral state can be conveyed to alveolar epithelial cells via macrophage-mediated paracrine signaling, resulting in substantial viral load reduction in human lung explants. These findings highlight a novel role for OMVs in fortifying mucosal immune barriers and establishing a transcellular antiviral network.

However, the acquisition of resistance genes can remodel the immunomodulatory properties of *E. coli* OMVs, effectively transforming them from immune activators into immunosuppressive agents (Figure 3b). Ye et al. demonstrated that *E. coli* OMVs carrying the MCR-1 resistance gene exhibit a tendency for immune evasion due to phosphoethanolamine modification of lipid A [82]. This modification reduces the affinity of LPS for the TLR4-MD-2 complex, thereby attenuating both the TLR4-mediated NF-κB signaling pathway and Caspase-11-dependent non-canonical inflammasome activation. Consequently, the secretion of key inflammatory cytokines is significantly diminished. Furthermore, these OMVs induce mitochondrial dysfunction and reactive oxygen species (ROS) accumulation, promoting macrophage apoptosis. In mouse models, animals injected with these OMVs developed milder systemic inflammation, indicating that the OMVs facilitate pathogen escape from immune surveillance via immunosuppression. While pathogenic *E. coli* OMVs typically coordinate both MyD88-dependent pro-inflammatory and TRIF-dependent antiviral responses via the TLR4 signaling axis, the acquisition of resistance genes can subvert this function, shifting the role of OMVs from immune activation to immune evasion. These findings deepen our understanding of OMVs immunomodulatory diversity and provide a conceptual framework for developing immune-based interventions against drug-resistant bacterial infections.

### E. coli OMVs-mediated antibiotic resistance

OMVs play a crucial role in the survival of Gram-negative bacteria. The increased vesiculation triggered by physical or chemical insults enhances bacterial fitness under these adverse conditions. For instance, under phage attack, bacteria release abundant OMVs that act as “decoys,” effectively neutralizing phages and thereby boosting population survival [83,84]. Furthermore, OMVs serve as highly efficient vectors for antibiotic resistance genes (ARGs), substantially expanding their potential for cross-species dissemination. Li et al. showed that subinhibitory concentrations of various antibiotics (e.g. gentamicin, meropenem, ciprofloxacin) promote the horizontal transfer of ARGs via a three-tiered cascade [85]. First, they activate the bacterial SOS response (e.g. approximately 30-fold upregulation of *recA*, approximately 10-fold of *lexA*), which potently stimulates OMV biogenesis. Second, this antibiotic stress not only increases OMV secretion but also elevates the copy number of carried *tet*(X4) resistance genes by approximately 20-fold. Third, the concomitant increase in OMV size and decrease in zeta potential collectively enhance the resistance of encapsulated DNA to nucleases. Alarmingly, unlike sporadic OMV-mediated resistance transfer observed in other species, *E. coli*, particularly the pandemic ST131 clone, has turned OMVs into an efficient, high-frequency dissemination network for *bla*_CTX-M-15_ and *mcr-1*, directly correlating with clinical treatment failures worldwide. Bielaszewska et al. found that OMVs can transfer plasmids harboring *bla*_CTX-M-15_ and *bla*_TEM-1_ to various Enterobacteriaceae, including nonpathogenic *E. coli*, *Salmonella*, and *Klebsiella* [86]. This contact-independent OMV-mediated gene transfer transcends species barriers, establishing persistent environmental reservoirs and enabling the trans-lineage dissemination of ARGs. This capacity poses a severe threat to clinical anti-infective therapy.

OMVs function not only as autonomous reservoirs for ARGs but also comprehensively enhance bacterial resistance through a tripartite mechanism encompassing genes, proteins, and biofilms. Carrera Páez et al. revealed a remarkable finding: even after the host bacterium lost the pDCAG1-CTX-M-15 plasmid, OMVs derived from *E. coli* ST615 continued to carry resistance genes such as *bla*_CTX-M-15_, *mcr-1*, and *aadB*, underscoring their capacity to serve as an ARG reservoir independent of the host’s genetic background [87]. Proteomic profiling further identified an enrichment of diverse resistance-related proteins in OMVs, including the MCR-1 enzyme, efflux pump components (AcrA, TolC), LPS assembly proteins (LptD, LptE), and stress response chaperones (DnaK, DegP). Protein–protein interaction network analysis delineated a synergistic resistance axis centered on MCR-1, which interconnected with the AcrAB-TolC efflux pump and 30S ribosomal proteins. Concurrently, the β-lactam resistance and antibiotic stress response pathways were enriched with 7 and 23 functional nodes, respectively. The research by Martínez et al. demonstrated that proteins within certain *E. coli* OMVs can directly execute resistance functions [61]. Specifically, OMVs from the EcNDM-1 strain carry the bNDM-1 carbapenemase, which degrades antibiotics and provides collective protection. Notably, resistance genes can themselves equip *E. coli* OMVs with novel immunomodulatory capabilities. For example, MCR-1-mediated lipid A modification enables OMVs to evade host immune recognition, thereby assisting bacterial populations in evading clearance (as detailed in Section 2.2) [82]. This reveals a novel OMV-mediated resistance strategy whereby bacterial survival is indirectly sustained through the active suppression of host immunity. In conclusion, *E. coli* OMVs play multifaceted roles in antibiotic resistance, with their biogenesis, composition, and functionality being intimately linked to this phenotype. Future studies should prioritize elucidating the precise mechanisms of OMV-mediated resistance to inform the development of novel antibacterial strategies.

### Other biological functions

In addition to the functions described above, *E. coli* OMVs play significant roles in two other domains. In reproductive health, Folliero et al. first established OMVs as direct effectors of bacterial-induced male infertility. Treatment of sperm with 8 μg/mL OMVs for 45 minutes reduced motility by 15%, concurrently increased ROS levels by 20%, and pushed the DNA fragmentation index above the clinical threshold. This damage was time-dependent [88]. The irreversible injury stems from OMV-induced mitochondrial dysfunction and DNA oxidative stress, providing a new etiological model for clinical cases of unexplained infertility. Regarding interspecies interactions, OMVs constitute a cross-kingdom regulatory network mediated by sRNAs. These sRNAs are selectively packaged into the vesicular lumen via the Hfq chaperone. This packaging strategy serves a dual purpose: it facilitates the silencing of host genes to augment virulence, while simultaneously protecting the sRNAs from nucleolytic degradation. Moreover, certain sRNAs, such as those involved in stress responses, can autoregulate their own biogenesis, thereby driving bacterial adaptive evolution [89]. These functions reveal OMVs as both potential disruptors of reproductive health and vital mediators of information exchange within microbial communities.

Collectively, the above sections illustrate the multifaceted roles of pathogenic *E. coli* OMVs in virulence, immune modulation, resistance dissemination, and reproductive toxicity. To provide a strain-specific summary, Table 1 compiles key findings from recent studies, highlighting how distinct pathotypes, serotypes, and resistance gene profiles shape OMV functional diversity. These findings offer a reference for future mechanistic and translational investigations.

**Table 1.** Pathogenic *E. coli* OMV-Mediated biological functions.

Strain	Pathotype / Serotype	Strain Characteristics (Virulence Factors/ Resistance Genes)	OMV-Mediated Biological Function	Ref.
*E. coli* 5791/99	EHEC; Serotypes O157:H7	Toxins: Stx2a, CdtV, EHEC-Hly, and H7 flagellin	Translocation across intestinal epithelial barrier; delivery of virulence factors; induction of transient barrier disruption	[77]
Pathogenic *E. coli*	None reported	Virulence factors present	Induces strong pro-inflammatory and antiviral responses; activates TLR1,3,4,5,6,7,8,9; upregulates IL-1, IL-6, IL-8, IL-12, TNF, IFN-α/β, IL-10	[80]
*E. coli* (#25922)	None reported	Virulence factors present	Inhibition of IAV and VSV replication; induces IFN-β and ISGs (IFIT1, IFI44 and Mx1) in macrophages	[81]
*E. coli* 47EC	None reported	Resistance genes: *tet(X4)*	Disseminating antimicrobial resistance	[85]
*E. coli* C227-11Φcu	EAEC; Serotypes O104:H4	ESBL phenotype; resistance genes: *dfrA7*, *sul2*, *strA*, *strB*, *tet(A)*, *bla*_CTX-M-15_ and *bla*_TEM-1_	Transfer of *bla*_CTX-M-15_, *bla*_TEM-1_, and pESBL plasmid via OMVs to various Enterobacteriaceae	[86]
*E. coli* M19736 (ST615)	None reported	resistance genes: *mcr-1*, *bla*_CTX-M-15_, *bla*_KPC-2_, *bla*_NDM-5_, *bla*_NDM-1_, *aadB*	Act as ARG reservoir; mediate spread of resistance determinants	[87]
*E. coli* ATCC 700,928	UPEC; Serotypes O6:H1	Fimbriae type 1 and P, non-hemagglutinin adhesin-siderophore receptor, salmonchelin siderophore receptor, alpha-hemolysin toxin, outer membrane protease T	Induces oxidative stress; reduces sperm motility; increases immobile sperm, ROS levels, and DFI	[78]

Abbreviations: EHEC: enterohemorrhagic *E. coli*; IAV: influenza A virus; VSV: vesicular stomatitis virus; ISGs: interferon-stimulated genes; EAEC: Enteroaggregative *E. coli*; UPEC: uropathogenic *E. coli*; ARG: antibiotic resistance gene; ROS: reactive oxygen species; DFI: DNA fragmentation index.

## Multifunctional applications of E. coli OMVs in biomedical platforms

The deepened understanding of the multifunctional nature of *E. coli* OMVs has prompted a shift in research focus from elucidating their pathogenic roles toward harnessing their therapeutic potential. By employing synthetic biology tools, these pathogen-derived OMVs are being engineered and repurposed, effectively shifting their role from vehicles of pathogenesis to versatile platforms for addressing infectious diseases and cancers.

### Application of E. coli OMVs in vaccines targeting multiple pathogens

Vaccines work by safely mimicking pathogens to activate both humoral and cellular immune responses, thereby preventing bacterial infections and associated diseases. OMVs, which combine inherent immunogenicity, low cytotoxicity, and high engineering plasticity, effectively circumvent pathogenicity, positioning them as a highly promising vaccine platform [12, 90, 91]. This is exemplified by the OMV-based vaccine MeNZB™ against *Neisseria meningitidis* serogroup B, which demonstrated approximately 70% real-world protective efficacy during outbreaks in New Zealand and Cuba [92]. It is noteworthy that the OMVs utilized in MeNZB™ have also been incorporated into the recently developed Bexsero® vaccine. In combination with three recombinant proteins (NHBA, NadA, fHBP) acting as adjuvants, it has been demonstrated to prevent Neisseria meningitidis serogroup B infection [35]. Addressing the shortage of vaccines against ETEC, Berzosa et al. demonstrated that ETEC-derived OMVs are promising vaccine candidates [93]. *In vitro*, ultracentrifugation-purified OMVs potently activated macrophages, eliciting a classic Th1/Th2-polarized response and boosting the secretion of key cytokines (IL-6, MCP-1, TNF-α, IL-12p70, IL-10) by approximately threefold. In murine models, immunization with these OMVs stimulated robust humoral immunity, marked by approximately 10-fold and 5-fold rises in serum IgG1 and IgG2a titers, respectively, and significantly reduced bacterial loads upon challenge, confirming their protective efficacy. Moreover, this study achieved a significant innovation in production technology by replacing conventional ultracentrifugation with a combined heat treatment and ultrafiltration method. This approach resulted in an approximate 15-fold increase in OMVs yield and a substantial reduction in production costs.

In addition to their successful application in bacterial vaccines, genetically engineered *E. coli* OMVs offer distinct advantages for viral vaccine development. They are inherently adjuvant, effectively activating innate immunity, and can be engineered to surface-display viral antigens, mimicking viral particles to enhance immunogenicity and delivery. Ninyio et al. exemplified this strategy by using CRISPR/Cas9 to precisely embed the HIV-1 gp41 MPER epitope into the OmpF protein of *E. coli* Nissle 1917 (EcN), enabling stable co-expression of the epitope on both the bacterial surface and its derived OMVs [94]. This approach not only ensured high-density antigen presentation (14.3 μg/10^8^ CFU) but, crucially, allowed the OMVs to function as “HIV-mimetic particles.” These particles promoted antigen recognition by dendritic cells and other immune cells, inducing combined systemic and mucosal immune responses to confer protective immunity. To further broaden the platform’s utility, Meenakshi et al. employed the Lpp-OmpA anchor to display the SARS-CoV-2 RBD protein in its native conformation on EcN OMVs [95]. The resulting RBD-OMVs induced high-titer specific IgG antibodies in mice, validating their potential as a COVID-19 vaccine candidate. Notably, the use of probiotic EcN as a chassis for OMV engineering is a paradigm shift. Unlike OMVs from attenuated *Salmonella* or *Shigella*, EcN is already considered safe for human consumption, significantly accelerating the regulatory pathway for oral OMV-based vaccines. Through surface display technologies (e.g. OmpF embedding, Lpp-OmpA anchoring), they present high-density viral antigens in native conformation, effectively mimicking whole viruses to provoke potent humoral immunity. These combined attributes underscore the broad application prospects of engineered OMVs as a versatile and potent vaccine platform.

### E. coli OMVs as transporters of biomolecules

Beyond vaccine development, *E. coli* OMVs have emerged as ideal delivery vehicles for bioactive molecules, leveraging their innate nanoscale size, excellent biocompatibility, and efficient delivery capabilities [96, 97]. For instance, Woo et al. used signal peptides to direct the localization of β-lactamase to the OMV surface and periplasm, creating nanoreactors with high stability against pH, temperature, and proteases [98]. This system degraded ampicillin and cefotaxime with 100-fold and 600-fold greater efficiency than the free enzyme, respectively, providing a scalable biological tool for controlling antibiotic resistance. In another study, Bittel et al. demonstrated the capacity of OMVs to deliver functional proteins and RNA across biological barriers [99]. Using *E. coli* engineered to express Cre recombinase and Rosa26.tdTomato reporter mice, they showed that in long-term colonization experiments, tdTomato expression was detected not only in intestinal crypt stem cells and mucosal macrophages but also extensively in distant organs-including the liver, kidneys, spleen, heart, and brain neurons-demonstrating their systemic delivery capability. This reveals the potential for microbiota to use OMVs as a vector for cross-species regulation of host gene expression.

The aforementioned studies underscore the versatility of OMVs as efficient, multifunctional platforms for biomolecule delivery. A critical next step for their clinical translation is achieving precise targeting. To this end, Liu et al. developed engineered OMVs based on the EcN strain, which displayed a ClyA – BMP-2/CXCR4 fusion protein, thereby achieving integrated bone tissue targeting and delivery of osteogenic factors [100]. In a murine model of ovariectomy-induced osteoporosis, these engineered OMVs effectively restored bone microarchitecture, enhanced bone mineral density, bone volume fraction, and trabecular number, and promoted bone formation by activating the BMP/SMAD signaling pathway. This scalable OMV platform offers an economical and efficient delivery strategy for anabolic bone therapy.

### Applications of E. coli OMVs in cancer therapy

In recent years, *E. coli*-derived OMVs have shown significant promise in cancer therapy. Their inherent immunostimulatory properties provide a unique advantage for remodeling the immunosuppressive tumor microenvironment (TME). Capitalizing on this, numerous current studies focus on engineering OMVs to achieve targeted delivery of tumor-specific antigens [101, 102]. For example, Laotee et al. utilized the SpyTag/SpyCatcher system to covalently display the MUC1-targeting single-chain antibody fragment scFv_SM3_ on the surface of *E. coli* OMVs. The resulting OMV – scFv_SM3_ complex specifically binds to and is internalized by MUC1-positive cancer cells [103], opening new avenues for the targeted delivery of chemotherapeutic drugs and nucleic acids. This surface engineering strategy has also been successfully applied to RNAi-based therapy. Gujrati et al. demonstrated that OMVs can serve as cell-specific siRNA delivery vehicles by displaying an anti-HER2 affibody on their surface. Systemic administration of OMVs loaded with siRNA against kinesin spindle protein resulted in targeted gene silencing, significant tumor growth inhibition, and minimal systemic toxicity, highlighting the potential of OMVs for RNAi-based targeted cancer therapy [104]. Beyond antigen targeting, functional modifications aimed at enhancing drug delivery efficiency and therapeutic efficacy are also being actively explored. Ren et al. innovatively encapsulated Polybia – mastoparan I fusion peptides within OMVs, constructing a dual-functional nanoplatform capable of both direct tumor killing and immune modulation [105]. In a bladder cancer model, this system effectively inhibited cell migration, promoted dendritic cell maturation, and enhanced cytotoxic T cell infiltration, demonstrating favorable therapeutic efficacy and safety. Similarly, Kan et al. developed an OMV system encapsulating hydrophobic titanium dioxide nanoparticles, which significantly increased ROS levels in oral squamous cell carcinoma cells, induced G2/M phase arrest, and enhanced radiotherapy efficacy by 2.5-fold [106]. These studies demonstrate that engineered OMVs hold considerable therapeutic value for enhancing local tumor killing and suppressing metastasis through diverse strategies, including targeted delivery, immune activation, and radiosensitization.

Despite the encouraging therapeutic outcomes achieved by the above engineered OMVs, the clinical application of naked OMVs via systemic administration remains severely limited by their dose-dependent toxicity. To overcome the dose-limiting systemic toxicity of naked OMVs, Feng et al. developed a radiation-controlled two-way adaptor by coating CD47 nanobody-displaying OMVs with a diselenide-containing PEG layer [107]. This surface modification increased the maximum safe intravenous dose by at least eightfold (from 100 μg to over 800 μg vesicle protein per mouse). Upon local tumor irradiation, the PEG layer was disrupted, releasing OMVs that simultaneously induced M1 polarization of tumor-associated macrophages and blocked the CD47-SIRPα signal, thereby triggering T cell-mediated antitumor immunity. This strategy achieved 100% long-term survival in a colon cancer model and established robust immune memory, conferring 100% tumor inhibition upon subcutaneous rechallenge and 98.2% inhibition of lung metastases. This work establishes a critical technical foundation: by overcoming the systemic toxicity bottleneck, it becomes feasible to safely administer OMVs intravenously, thereby unlocking their potential for combination with a wide range of immune-based therapies.

Building on this ability to safely deliver OMVs systemically, researchers have further exploited their intrinsic immunostimulatory properties to synergize with immune checkpoint blockade (ICB) therapy, addressing the critical challenge of poor T cell infiltration and an immunosuppressive TME that limit the efficacy of current ICB agents. One pioneering strategy involves engineering OMVs to directly block the PD-1/PD-L1 axis while retaining their intrinsic immunostimulatory properties. Li et al. genetically fused the programmed death-1 (PD-1) ectodomain to *E. coli* outer membrane vesicles (OMV-PD1) [108]. These engineered vesicles not only promoted pro-inflammatory cytokine secretion but also bound to tumor PD-L1, inducing its internalization and degradation, thereby relieving T cell suppression. In murine CT26 (colorectal) and B16 (melanoma) models, OMV-PD1 treatment significantly enhanced CD8^+^ T cell infiltration and reduced tumor weight by 6–7 fold vs controls. Notably, OMV-PD1 achieved 50% complete tumor regression, with 66.7% of cured mice rejecting tumor rechallenge, outcomes that were superior to those of anti-PD-L1 antibody alone. Furthermore, Won et al. demonstrated that mass-produced *E. coli* OMVs alone, without any antigen engineering, could activate cancer antigen-specific stem-like CD8^+^ T cells characterized by TCF-1 and PD-1 co-expression, effectively converting “cold” tumors into “hot” tumors [109]. Specifically, intratumoral administration of OMVs significantly increased tumor-infiltrating CD8^+^ T cells and neoantigen-specific CD8^+^ T cells by several folds compared to vehicle control. Moreover, OMVs alone induced a marked increase in TCF-1^+^ stem-like CD8^+^ T cells, which are known to respond to immune checkpoint blockade. When combined with anti-PD-1 antibody, these OMVs exhibited synergistic antitumor activity: combination therapy reduced tumor volume significantly more than either monotherapy, accompanied by enhanced infiltration of proliferating CD8^+^ T cells and increased expression of cytotoxic molecules within the tumor microenvironment. OMVs remodel the TME by activating innate immunity and promoting T cell infiltration, while ICB relieves the suppressive signals that limit T cell effector function. This synergistic combination is particularly potent against poorly immunogenic or checkpoint inhibitor-resistant tumors.

The aforementioned research solidifies the considerable value of engineered OMVs as a local therapeutic platform. More remarkably, scientists are now aiming to transform them from local tools into “immunovaccines” capable of provoking systemic anti-tumor immunity. A critical advancement in this transition was achieved by Yue et al. [110]. In their study, mice were orally administered arabinose along with engineered bacteria capable of expressing tumor antigen-mFc fusion proteins. In the gut, arabinose induced these bacteria to produce and secrete OMVs displaying tumor antigens (OVA or Adpgk) on their surface, which ultimately initiated a specific anti-tumor immune response. In murine models of melanoma lung metastasis and subcutaneous colon cancer, OMV-OVA-mFc and OMV-Adpgk-mFc markedly reduced the metastatic burden and suppressed tumor growth by approximately 80%, respectively. Furthermore, the proportion of effector memory T cells in the spleens of immunized mice remained persistently elevated, establishing long-term immune memory and conferring potent resistance to tumor rechallenge. The study also revealed that while direct injection of these OMVs into the colon was effective, oral administration of pre-formed OMVs was not, underscoring the necessity for in situ OMV production by the bacteria. Importantly, the engineered bacteria were cleared within 24 hours post-administration, thereby presenting no colonization risk and offering a novel strategy for systemic OMV-based immunotherapy.

The developed platform underscores the considerable potential of *E. coli* OMVs as multifaceted tools in modern medicine. To delineate the scope and efficacy of these applications, Table 2 systematically categorizes and contrasts the key methodologies and their representative outcomes across the fields of pathogenic vaccination, biomolecular delivery, and oncology [111–120].

**Table 2.** Applications of engineered *E. coli* OMVs in biomedical platforms.

Strain	Engineering Strategy	Application Field	Target Pathogen/Disease /Study Focus	Displayed/Delivered Component	Key Function	Ref.
*E. coli* Nissle 1917	Vaccine	CRISPR/Cas9-mediated insertion of HIV-1 MPER epitope into the *ompF* gene.	HIV-1	HIV-1 gp41 MPER epitope	Stable surface expression of MPER epitope over 30 passages, recognized by anti-HIV-1 gp41 (2F5) antibody in both live and heat-killed bacteria.	[94]
*E. coli* Nissle 1917	Vaccine	Lpp-OmpA surface display system to fuse and express the RBD.	COVID-19	ARS-CoV-2 Spike protein RBD	Efficient expression of native RBD.	[95]
*E. coli* Top10	Vaccine	Expression of ClyA – tumor antigen – mFc fusion protein under Ara-inducible promoter.	Pulmonary metastatic melanoma (B16-OVA) and subcutaneous colon cancer (MC38)	Tumor antigens (OVA/ Adpgk) fused to ClyA and mouse IgG Fc fragment	Induces antigen-specific cytotoxic T lymphocytes, inhibits tumor growth, and establishes long-term immune memory.	[110]
*E. coli* BL21	Vaccine	PelB signal peptide for luminal localization; Lpp-OmpA hybrid for surface display.	EHEC	Int280	Induction of anti-intimin IgG response.	[111]
*E. coli* Nissle 1917 (Δ*flhD*)	Vaccine	Flagella-deficient probiotic derivative	Periodontitis	None	Acts as a potent mucosal adjuvant to enhance humoral immune responses.	[112]
*E. coli* BL21 and Δ*ompA* mutant	Vaccine	Fusion of F1V to OmpA leader sequence or transmembrane domain	Plague	F1V fusion protein	Induced higher serum anti-LcrV and anti-F1 IgG titers; elicited a balanced Th1/Th2 immune response.	[113]
*Escherichia coli* DH5α	Vaccine and cancer therapy	HM-NPs composed of EM and autologous TM coated onto PLGA nanoparticles.	CT26 colon cancer, 4T1 breast cancer, B16-F10 melanoma, EMT6 breast cancer	Tumor antigens from TM and bacterial components from EM.	Enhances dendritic cell maturation, activates T cells, induces tumor regression, prevents recurrence.	[114]
*E. coli* BL21	Vaccine and cancer therapy	Surface modification with maleimide.	Colon cancer	Inhibitor of IDO	IDO inhibition, the immunosuppressive microenvironment overcoming.	[115]
*E. coli* DH5α	Biomolecule Delivery	Expression of Cre-recombinase via plasmid vector.	Microbe-Host communication	Cre protein and Cre RNA	Transfer of functional biomolecules to host cells, inducing Cre-LoxP-mediated gene recombination and proinflammatory responses.	[99]
*E. coli* Nissle 1917	Biomolecule Delivery	Construction of recombinant plasmid pClyA-BMP-2-CXCR4; fusion of BMP-2 and CXCR4 with OMVs surface protein ClyA.	Osteoporosis	BMP-2 and CXCR4	Promotion of osteogenic differentiation and inhibition of adipogenic differentiation of BMSCs; amelioration of osteoporosis in OVX mouse model via BMP/SMAD signaling pathway.	[100]
*E. coli* (E44 and DH5α)	Biomolecule Delivery and cancer therapy	E44- and DH5α-derived, GRP94 binding.	Brain metastases	Dexamethasone and embelin	Crosses BBB and regulate the serpin B2 and neuroserpin; restores plasmin production; inactivates L1CAM.	[116]
MG1655	Biomolecule Delivery and cancer therapy	Surface modification with maleimide.	B16F10 melanoma, CT26 colon cancer	MerTK inhibitor	Inhibits efferocytosis by blocking MerTK phosphorylation in TAMs, promotes secondary necrosis of tumor cells, captures and delivers TAAs to lymph nodes, activates dendritic cells and CD8^+^ T cells, induces antitumor immunity.	[117]
*E. coli* BL21	Cancer therapy	SpyTag/SpyCatcher system with Lpp’OmpA-SpyCatcher fusion on OMVs and SpyTag-scFvSM3 from CHO cells.	MUC1-expressing cancer cells	Anti-MUCI scFvSM3	Specific binding and internalization into MUC1-positive cells.	[103]
*E. coli* K-12 W3110 *msbB* mutant	Cancer therapy	ClyA-affibody fusion surface display; *msbB* mutation for penta-acylated LPS	HER2-overexpressing cancers (SKOV3, BT474, HCC-1954)	Anti-HER2 affibody; KSP siRNA	HER2-specific targeting, KSP silencing, mitotic arrest, apoptosis, tumor growth inhibition.	[104]
*E. coli* BL21	Cancer therapy	Construction of recombinant plasmids (pET43.1a-MPI-N, pET43.1a-MPI-C, pET43.1a-MPI-SP) for fusion peptide expression; use of OmpA signal peptide to enhance encapsulation.	Bladder cancer	MPI fusion peptides (MPI-C, MPI-N, MPI-SP)	Inhibition of bladder cancer cell (MB49, UMUC3) proliferation/migration and induce apoptosis; activation of antitumor immune response.	[105]
*E. coli* DH5α	Cancer therapy	Encapsulation of TiO_2_ nanoparticles within OMVs via high-frequency ultrasonication.	Oral squamous-cell carcinoma	TiO_2_ nanoparticles	Enhanced radiosensitization and immune activation; increased ROS production; selective cytotoxicity to cancer cells	[106]
*E. coli* BL21 (DE3)	Cancer therapy	Fusion of CD47nb with surface protein ClyA; metabolic labeling with Ac4GalNAz; click chemistry with PEG/Se for radiation-triggered release	MC38-OVA colon cancer, B16-OVA melanoma	CD47 nanobody	Blocks CD47 “don’t eat me” signal, induces M1 polarization, bridges tumor cells and macrophages, enhances phagocytosis, remodels TME, activates T cell immunity and long-term memory.	[107]
*E. coli* W3110 (Δ*msbB*)	Cancer therapy	Surface display of PD1 ectodomain via genetic fusion to outer membrane protein ClyA.	B16 melanoma, CT26 colorectal cancer	Mouse PD1 ectodomain	Activates DC maturation and NK cells; induces pro-inflammatory cytokines; binds PD-L1 on tumor cells and promotes its lysosomal degradation; protects CD8^+^ T cells from exhaustion; enhances T cell infiltration; suppresses tumor growth and induces immune memory.	[108]
*E. coli* BL21 (DE3) Δ*msbB*	Cancer therapy	*msbB* knockout; metal precipitation and size-exclusion chromatography.	MB49, EMT6, MC38-OVA, B16F10-OVA tumor models	None	Induces tumor regression, activates cancer antigen-specific stem-like CD8^+^ T cells, synergizes with anti-PD-1.	[109]
*E. coli* BL21	Cancer therapy	CRISPR/dCas9 system fused with outer membrane protein ClyA via sgRNA-mediated targeting.	MB49 bladder cancer, B16F10 melanoma, and MDA-MB-231 breast cancer	Plasmid DNA encoding CXCL9 and IL12	Enhances T cell chemotaxis and activation in tumor microenvironment.	[118]
*E. coli*	Cancer therapy	High-pressure co-extrusion of OMVs with MSN-5-FU-HA.	Colon cancer	5-Fluorouracil	Targeted drug delivery to colon cancer cells, reduced systemic toxicity, and improved drug release in tumor microenvironment.	[119]
*Escherichia coli* BL21	Cancer therapy	CRISPR-mediated gene knockout of *msbB* to reduce endotoxicity.	Triple-negative breast cancer	Redd1 siRNA; Paclitaxel; mannose ligand	Redd1 knockdown in TAMs to enhance glycolysis and repolarize macrophages to M1 phenotype; increased CD8^+^ T cells, mature dendritic cells, decreased Tregs.	[120]

Abbreviations: MPER: membrane-proximal external region; RBD: receptor binding domain; HM-NPs: hybrid membrane nanoparticles; EM: *E. coli* cytoplasmic membranes; TM: tumor cell membranes; IDO: indoleamine 2, 3-dioxygenase; BMSCs: bone marrow mesenchymal stem cells; BBB: blood-brain barrier; CAM: cell adhesion molecular; MerTK: myeloid-epithelial-reproductive tyrosine kinase; TAAs: tumor-associated antigens; MPI: polybia – mastoparan I. TAMs: tumor-associated macrophages.

### Core challenges in the clinical application of E. coli OMVs

Despite the considerable promise of *E. coli*-derived OMVs in vaccine development, drug delivery, and cancer therapy, their clinical translation faces several critical challenges. Safety remains a fundamental hurdle: OMVs from pathogenic strains contain endotoxin components such as lipid A, which potently activate the TLR4-MD-2 pathway. This triggers both NF-κB signaling and the caspase-11-dependent non-canonical inflammasome pathway. While this property underlies their strong adjuvant effect, it concurrently carries the risk of provoking excessive inflammation or even systemic toxicity [82]. Manufacturing presents another obstacle; the intricate OMV extraction process leads to significant batch-to-batch variation, making stable, large-scale production difficult to achieve [121]. Quality Control is also a major constraint, as precise command over critical quality attributes – including drug-loading efficiency and the encapsulation stability of functional molecules – remains inadequate, severely hampering the development of standardized formulations [122]. Finally, the Pharmacokinetic Profile of native OMVs is suboptimal: they lack inherent targeting capacity and are rapidly cleared by the mononuclear phagocyte system. Although engineering surface modifications can enhance targeting, these strategies still grapple with short circulation half-lives, low tumor accumulation efficiency, and significant off-target effects [123]. Nevertheless, these impediments are largely technical and do not undermine the core advantages and immense potential of OMVs as innate delivery and adjuvant platforms. Encouragingly, as research advances, key issues such as directed antigen display and precise immunogenicity modulation are being progressively addressed. We can thus anticipate that with continued breakthroughs in engineering strategies and production processes, the highly promising *E. coli* OMV platform is poised to make significant contributions to the advancement of novel vaccines and cancer immunotherapies.

## Conclusion

The multifaceted role of *E. coli* OMVs in pathogenesis and resistance underscores their significance not only as fundamental biological entities but also as a platform for innovative biomedical applications. Looking ahead, a translational medicine revolution centered on engineered OMVs is rapidly gaining momentum. In vaccinology, their triple synergistic advantages – inherent adjuvanticity, efficient nanodelivery, and lyophilization stability – are poised to overcome critical accessibility hurdles globally. Simultaneously, in oncology, strategies employing localized targeting, combined immunoradiotherapy, and oral lifelong immunization are ushering in a new era of mucosal immunity against cancer.

Despite the promise, the clinical translation of *E. coli* OMVs faces persistent challenges, including the need for precise tissue targeting, a balanced immunogenic profile, and scalable manufacturing of uniform vesicles. The path forward lies in the rational design of synthetic OMV platforms. This entails developing precision targeting strategies, such as ligand-receptor display or smart membrane modifications, to maximize on-target delivery and minimize off-target effects. Equally critical is the fine-tuning of immunogenicity via lipid A engineering or co-delivery of immunomodulators to harness adjuvant potency while averting harmful inflammation. The convergence of nanotechnology with synthetic biology is key to this endeavor, enabling the creation of artificial OMVs using tools like orthogonal gene circuits for controlled cargo loading, CRISPR-Cas editing for precise composition control, and AI-assisted antigen optimization. We anticipate that these next-generation OMVs, engineered through such targeted and programmable strategies, will break through the bottlenecks of conventional interventions. They hold the potential to establish powerful new paradigms in anti-infection therapy, anti-tumor strategies, and drug resistance management, ultimately offering transformative solutions to pressing global public health crises.

## Data Availability

Data sharing are not applicable – no new data are generated.
